# Convolutional neural networks improve fungal classification

**DOI:** 10.1038/s41598-020-69245-y

**Published:** 2020-07-28

**Authors:** Duong Vu, Marizeth Groenewald, Gerard Verkley

**Affiliations:** Westerdijk Fungal Biodiversity Institute, Uppsalalaan 8, 3584CT Utrecht, The Netherlands

**Keywords:** Classification and taxonomy, Machine learning, Metagenomics

## Abstract

Sequence classification plays an important role in metagenomics studies. We assess the deep neural network approach for fungal sequence classification as it has emerged as a successful paradigm for big data classification and clustering. Two deep learning-based classifiers, a convolutional neural network (CNN) and a deep belief network (DBN) were trained using our recently released barcode datasets. Experimental results show that CNN outperformed the traditional BLAST classification and the most accurate machine learning based Ribosomal Database Project (RDP) classifier on datasets that had many of the labels present in the training datasets. When classifying an independent dataset namely the “Top 50 Most Wanted Fungi”, CNN and DBN assigned less sequences than BLAST. However, they could assign much more sequences than the RDP classifier. In terms of efficiency, it took the machine learning classifiers up to two seconds to classify a test dataset while it was 53 s for BLAST. The result of the current study will enable us to speed up the taxonomic assignments for the fungal barcode sequences generated at our institute as ~ 70% of them still need to be validated for public release. In addition, it will help to quickly provide a taxonomic profile for metagenomics samples.

## Introduction

Microbes are essential for all life forms on Earth. It is crucial to understand these complex communities as they are key players in maintaining environmental stability. So far, the study of microbial communities has focused primarily on prokaryotes. However, fungi are eukaryotic microorganisms that play fundamental ecological roles as decomposers, symbionts, mutualists, and pathogens. Despite their enormous diversity and importance in ecosystems, we lack the knowledge about the general pattern of fungal diversity and their functional roles in the environment. The rapid development of sequencing technologies has enabled us to investigate microbes in their natural environments using a metagenomics approach. Environmental samples from natural communities are collected, and bulk DNA is extracted and sequenced using high throughput sequencing technologies. The metagenomics approach to study fungal communities targets specific genes, the so-called barcodes, to provide a taxonomic profile of diversity of the environmental samples^1–3^. The Internal Transcribed Spacer (ITS) region was proposed as a universal barcode for fungi^4^. The generated DNA sequences are clustered into Operational Taxonomic Units (OTUs) with a given threshold for species identification. Representative sequences of the OTUs are classified against reference sequences. The most common approach for sequence classification is based on BLAST^5^, which assigns sequences to the group of their best match if the obtained similarity score is high enough.

There are a number of challenges in sequence classification. The first problem is the lack of reference sequences. Less than 1% of the estimated 3.8 million species^6^ of fungi have ITS sequences available in GenBank and many of the sequences are often of poor quality^7,8^. At the Westerdijk Fungal Biodiversity Institute, Utrecht, The Netherlands, more than 100,000 living fungal strains are preserved that were originally assigned to ca. 17,000 species. When accessioned, each identified strain is assigned a taxon name from MycoBank^9^, an online registration system for fungal species and higher level taxon names. Being one of the largest fungal culture collections in the world, we have generated more than 200,000 DNA barcode sequences for fungal identification in a DNA barcoding project^10^. A large number (~ 30,000) of fungal barcodes, in which every sequence was manually validated by experts, has recently been released for public use in^8,11^.

The second problem is that the current fungal taxonomic classification is imbalanced because of clade-specific evolutionary histories. In addition, the continuous development in fungal taxonomy results in an on-going stream of reclassifications and introduction of new names, making informed decisions about fungal taxonomic delineation highly uncertain, compared to bacteria^12,13^. In most fungal ecology studies, a threshold of 97% is given as default for species identification. This threshold is rather low for bacterial and fungal species identification^8,11,12^ using 16S and ITS sequences. We have recently proposed a method to predict an optimal threshold for taxonomic delineation^14^. Based on our released barcode datasets, optimal thresholds predicted for yeast and filamentous fungal (mold) identification at the species levels could achieve a high accuracy of ~ 80%^8,11^. However, at higher taxonomic levels, the obtained accuracy scores were still low.

Finally, the main bottleneck in sequence classification is the comparison of the representative sequences in metagenomics samples with reference sequences. Although BLAST has been shown to be efficient, aligning hundreds of thousands to millions of sequences to the reference sequences still poses a computational challenge as DNA sequence alignments are computationally expensive^15^.

Machine learning has been proposed in metagenomics for rapid taxonomic assignments in bacteria^16–18^. The Ribosomal Database Project (RDP) Bayesian classifier^18^ was applied in many metagenomics studies^19–21^ for bacteria. It was also adopted for fungal classification in^22^ using 28S rRNA large subunit, in^23^ using 18S rRNA small subunit, and recently in^24^ using ITS sequences. The RDP classifier has been shown to be accurate for fungal classification as the accuracies of sequence assignment at the genus level were quite high, ranging from 80 to 90%^22–24^. Deep learning has recently emerged as a successful paradigm for big data classification and clustering^25^. Next to successful applications in image and natural language analysis, it has started offering data-driven solutions to sequence-based problems in genomics sequence analysis^26,27^. Deep learning has been applied for bacterial taxonomic classification on a dataset of simulated 16S DNA sequences^28^. This approach seems promising as the results obtained using two different models, convolutional neural network (CNN)^25^ and deep belief network (DBN)^29,30^ outperformed the RDP Bayesian classifier.

In this paper, we apply CNN, DBN, RDP and BLAST to our recently released yeast and mold barcode datasets^8,11^ to find the best method for fungal classification. We also reclassify a novel dataset, the “Top 50 Most Wanted Fungi”^31^ which was compared with the mold dataset in^11^, using the yeast barcode dataset for the evaluation. The result of the current study will enable us to speed up the taxonomic assignments for the fungal barcode sequences generated at our institute as ~ 70% of them still need to be validated for public release. In addition, it will help to provide quickly a taxonomic profile for metagenomics samples.

## Results

### Evaluation of the barcode datasets

This section evaluates the performance of CNN, DBN, RDP and BLAST on the yeast dataset. The evaluation of the classifiers on the mold dataset can be found in the Supplementary File. The yeast dataset consisted of 3,784 ITS sequences representing 61% cultured yeasts that was used in^32^ and released as a subset in (^8^, https://www.ncbi.nlm.nih.gov/bioproject/PRJNA351778) in which taxa at all taxonomic levels were available and downloaded from MycoBank^9^. In total, there were 1,035 species, 138 genera, 45 families, 18 orders, and 9 classes. The performance of the CNN and DBN classifiers were evaluated with sequences being represented as *k*-mer frequency vectors with *k* = 4, 6, and 8. The dataset was split into two datasets, the training and test datasets, in a tenfold cross-validation procedure. The evaluation of the classifiers at a taxonomic level was performed on the same training and test datasets. On average, there are 3,406 (90%) sequences of 971 (94%) species, 135 (98%) genera, 45 (100%) families, 18 (100%) orders, and 9 (100%) classes for training and 378 sequences for testing of which 64 (1.7%), 3 (0.08%), 0, 0, and 0 sequences in the test dataset had no labels in the training dataset at the species, genus, family, order, and class level respectively.

#### The distribution of the barcodes

**Figure 1:**
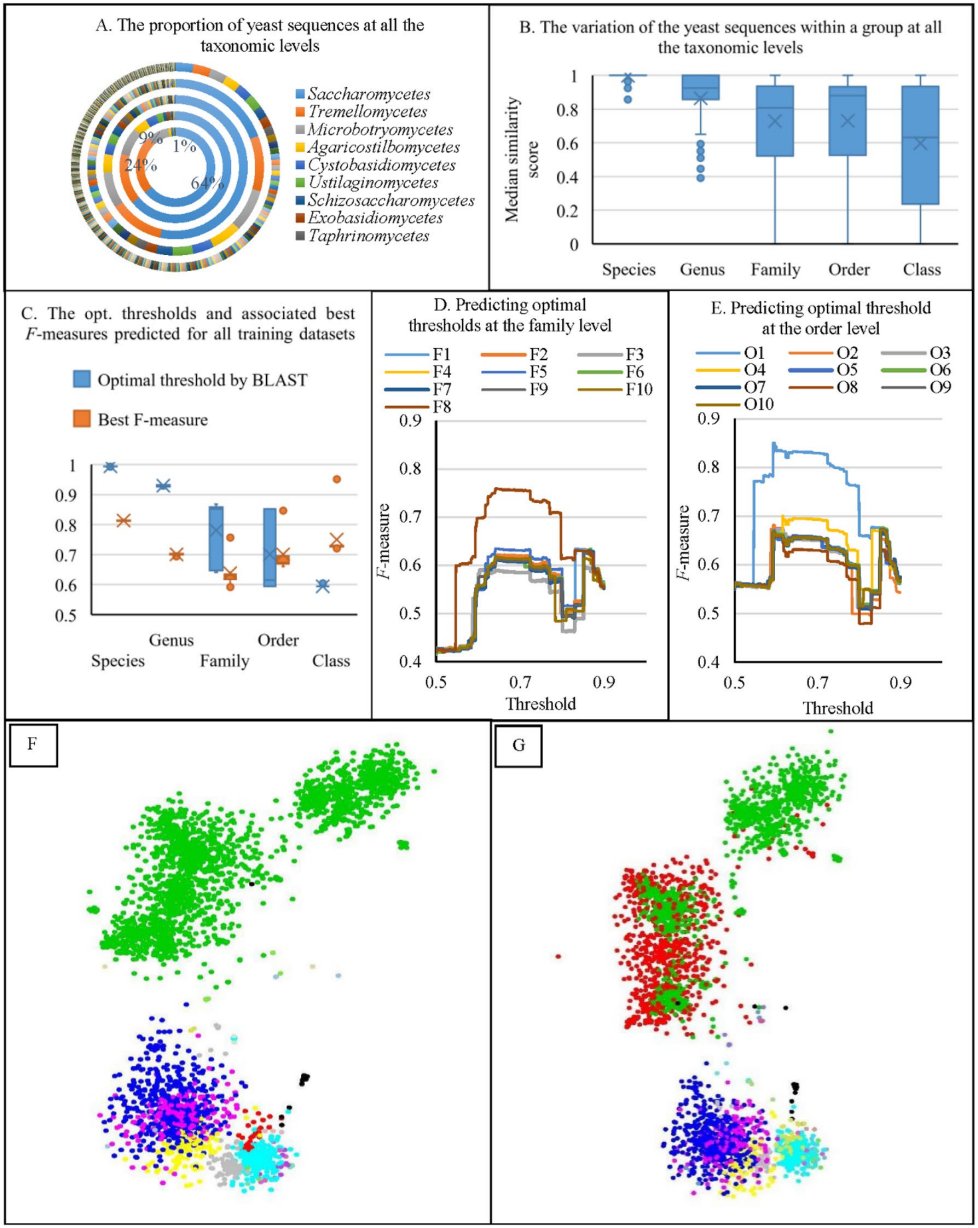
(**A**) The proportion of yeast sequences at all taxonomic levels. The smallest ring represents the class level, followed by the order, family, genus and species levels. (**B**) The variation of the median similarity scores of the yeast groups at all taxonomic levels. (**C**) The optimal thresholds and the associated best *F*-measures predicted for all yeast training datasets at all taxonomic levels. (**D**) Predicting optimal thresholds for the yeast training datasets using a series of thresholds (between 0.5 and 0.9, with a step of 0.001) at the family level. (**E**) Predicting optimal thresholds for the yeast training datasets using a series of thresholds (between 0.5 and 0.9, with a step of 0.001) at the order level. (**F**) The distribution of the yeast dataset. The sequences were colored based on the order name. The sequences of the largest order *Saccharomycetales* (2,427) were in green, followed by *Tremellales* (559) in blue, *Sporidiobolales* (305) in cyan, *Trichosporonales* (159) in pink, *Filobasidiales* (122) in yellow, etc. The coordinators of the sequences were generated using fMLC^32^. The sequences were visualized using the rgl package in R (https://r-forge.r-project.org/projects/rgl/). The numbers in brackets are the numbers of the sequences in the current group. (**G**) The sequences were colored as in (**F**) except that the sequences of the *Candida* genus (730) were colored in red.

Initially, we studied the distribution of the yeast barcodes to evaluate classification results. Figure 1A shows the proportion of the barcodes at each taxonomic level. It can be seen that the sequences were not equally distributed. The largest group (*Saccharomycetes*) at the class, order, family and genus levels consisted of 64%, 64%, 54%, and 19% of the sequences of dataset, respectively. The median similarity scores of sequences within a group at each level were also varied, specifically at higher taxonomic levels, as seen in Fig. 1B. To determine the reasonable similarity score for separating the sequences at different taxonomic levels, we clustered the sequences to find the best match to the current taxonomic classification. Figure 1C shows the optimal thresholds and the associated best *F*-measures predicted for all the training datasets at all taxonomic levels. At the species, genus and class levels, the predicted thresholds were consistent, which were ~ 99.4%, ~ 93%, and ~ 59.4%, respectively. At the family and order levels, the ranges of the predicted thresholds were large from ~ 60 to ~ 85%. We took the predicted results at the family (Fig. 1D) and order (Fig. 1E) levels for further investigation. Figure 1D,E show a bimodal distribution suggesting two optimal thresholds of ~ 60% and 85% with the highest *F*-measures for yeast classification at the family and order levels. It is because these training datasets contained the largest yeast order *Saccharomycetales* and family *Saccharomycetaceae* in which the barcode sequences were highly variant and being split into two groups as can be seen in Fig. 1F. The largest genus, *Candida* (Fig. 1G, in red) within the order *Saccharomycetales* has been suggested for reclassification in^8^. Except for the species level having a high clustering accuracy of 81%, the accuracies of predicting optimal thresholds to classify the sequences at the higher taxonomic levels were low, ~ 70%, 63%, 70% and 75% for the genus, family, order and class levels, respectively (see Fig. 1C). For the mold dataset, similar results were observed (see Supplementary Section 2). Although our barcodes datasets were not complete, these results still indicated an imbalance problem for fungal references. The thresholds predicted for sequence discrimination in this section were different from the thresholds predicted in^8,11^. In^8,11^, at the species level, the predicted threshold was 98.41% with a high *F*-measure of 90.67% for yeasts, and 99.61% with an *F*-measure of 84% for molds. It is because the species sharing the same ITS sequences (~ 6% for yeasts and 17% for molds) were excluded from these predictions. At the genus level, the predicted threshold was 96.31% with an *F*-measure of 61% for yeasts, and 94.31% with an *F*-measure of 64% for molds. At the higher taxonomic levels, no prediction was made for yeasts. For molds, the thresholds to discriminate families, orders, and classes were 88.51%, 81.21%, and 80.91% with low *F*-measures of less than 60%. Although the datasets used for the predictions in^8,11^ were larger (with ~ 1,500 yeast and ~ 3,200 mold species), the obtained low *F*-measures still revealed the need for a revision of fungal classification at higher taxonomic levels. This result illustrated a problem of uncertainty in making informed decisions about fungal taxonomic delineation as different groups of fungi, different datasets, and different alignment programs will produce different optimal thresholds for sequence discrimination.

#### The quality of the classifiers

**Figure 2:**
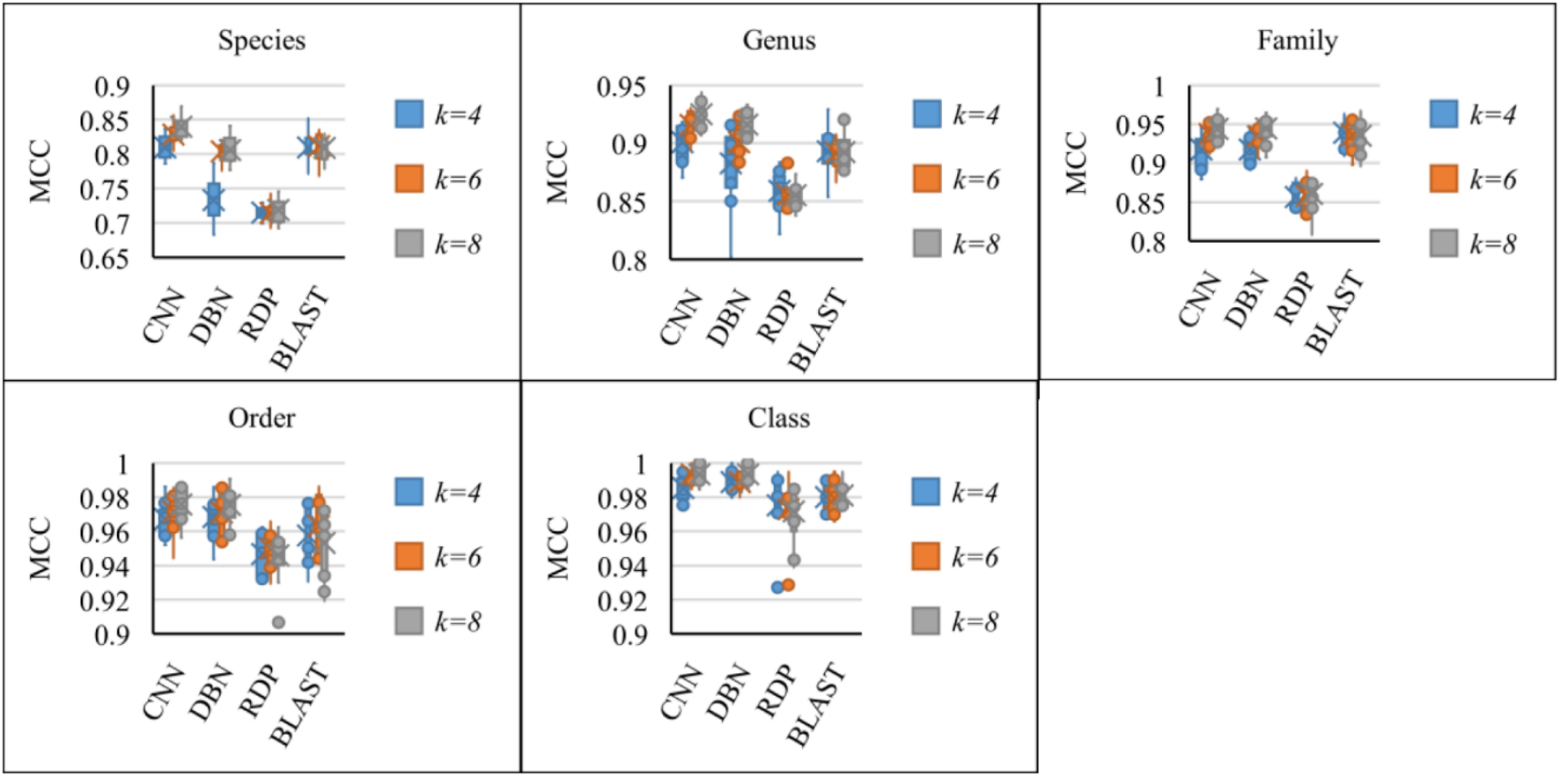
The MCCs obtained by different classifiers at different taxonomic levels for *k* = 4, 6, and 8.

To compare the qualities obtained by CNN, DBN, RDP and BLAST, we used the Matthews correlation coefficient (MCC)^33^ as a performance measure as it takes into account true and false positives which can be used even if the classes are of very different sizes. MCC was initially designed for binary classifications, and has been generalized to the multiclass case^34^. Other performance merits such as accuracy, precision, recall, and confusion matrices can be found in the Supplementary File. Only the test sequences with a label in the training dataset were taken into account. Figure 2 and Table 1 show the range and average of the obtained MCCs at different taxonomic levels. It can be seen that the MCCs varied up to 5%, except for the DBN classifier at genus level when *k* = 4. On average, the MCCs of CNN and DBN increased slightly up to 1% at all taxonomic levels when *k* = 8 compared with when *k* = 6. When *k* = 4, the MCC obtained by DBN was low at the species level which were ~ 7% less than the one of CNN.

**Table 1:** The average MCCs obtained by different classifiers at different taxonomic levels with *k* = 4, 6, and 8.

Level	*k*	CNN	DBN	RDP	BLAST
Species	4	**0.8093**	0.7331	0.7146	**0.8093**
	6	**0.8284**	0.8040	0.7145	0.8112
	8	**0.8394**	0.8056	0.7185	0.8086
Genus	4	**0.9007**	0.8828	0.8588	0.8931
	6	**0.9157**	0.9010	0.8555	0.8917
	8	**0.9249**	0.9159	0.8550	0.8925
Family	4	0.9172	**0.9176**	0.8567	0.9387
	6	**0.9374**	0.9343	0.8579	0.9360
	8	0.9438	**0.9446**	0.8602	0.9350
Order	4	0.9672	**0.9683**	0.9466	0.9575
	6	**0.9716**	0.9708	0.9497	0.9630
	8	**0.9757**	0.9753	0.9460	0.9531
Class	4	0.9853	**0.9889**	0.9753	0.9801
	6	**0.9934**	0.9889	0.9729	0.9792
	8	**0.9934**	**0.9934**	0.9716	0.9811

The highest average MCCs for each *k* at all taxonomic levels are highlighted in bold.

The CNN classifier outperformed the other classifiers at most of the taxonomic levels. Although the DBN classifier produced a low accuracy score at the species level, at the higher taxonomic levels, it produced a similar MCC as the CNN classifier. The BLAST classification produced a slightly lower (less than 1%) MCC value than the CNN classifier, while RDP classifier produced the lowest MCCs at all taxonomic levels. At the genus and higher taxonomic levels, all the classifiers achieved a high MCC value of more than ~ 85%. For the mold dataset, again the CNN classifier outperformed the other classifiers (see Supplementary Fig. 7E). BLAST classification produced the lowest MCCs at all taxonomic levels as the optimal thresholds predicted for this dataset excluded all the sequences lying in the border of the groups. One of the reasons that the qualities of the species classification were low is that ~ 6% and ~ 17% of the yeast and mold species shared the same ITS barcodes^8,11^.

#### The efficiency of the classifiers

Table 2 shows the average time required for the training and testing of the BLAST, RDP, CNN, and DBN classifiers. The training time for BLAST was the time needed for finding an optimal threshold for classification. It can be seen that except for the BLAST classification, all the other classifiers were efficient in classifying, taking them less than two seconds to classify these datasets. The RDP trained faster than the other classifiers. The CNN and DBN classifiers were rather efficient when *k* was set to 4. When *k* was set to 6, it took the CNN (DBN) classifier 20 (9) minutes, while it took the BLAST classification 34 min to train each of the training datasets. The CNN and DBN classifiers were extremely slow when *k* was set to 8, requiring approximately five and one hours to train each of the training dataset, respectively.

**Table 2:** Average run-time performance of all classifiers on each of the training dataset.

Classifier	Training time (s)	Classifying time (s)
DBN (*k* = 4)	289.25	**0.18**
DBN (*k* = 6)	558.55	0.23
DBN (*k* = 8)	3,788.01	0.99
CNN (*k* = 4)	190.25	0.27
CNN (*k* = 6)	1,196.72	0.52
CNN (*k* = 8)	19,399.33	5.10
RDP	**1.58**	1.40
BLAST	2057	53.67

The most efficient run-time performances for training and testing are highlighted in bold.

Based on our comparisons on accuracy and efficiency, *k* = 6 is the best option for CNN and DBN as the classification quality scores reduced slightly but the run-time performance improved significantly when comparing with *k* = 8. Among the classifiers, the CNN classifier with *k* = 6 is best choice for fungal classification. Although, it was not the best in terms of efficiency, it took only 20 min to train and 0.52 s to classify the test datasets. In terms of quality, it improved up to 2% for yeast classification at all taxonomic levels, compared with the traditional BLAST classification.

#### Classification results

**Figure 3:**
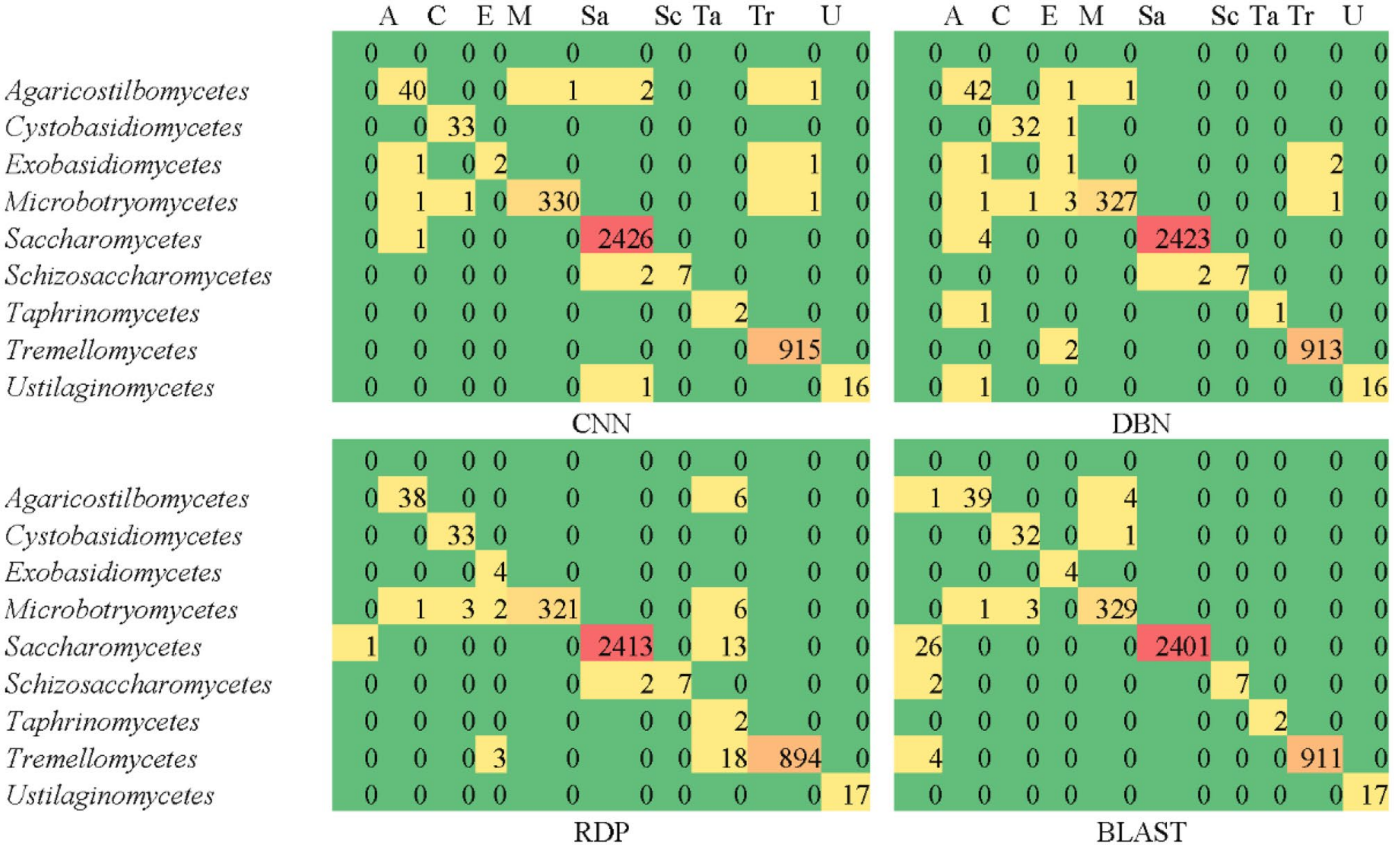
The confusion matrices obtained by all the classifiers at the class level.

To explore how the yeast taxa were handled by CNN, DBN, RDP and BLAST individually, we took the classified results when *k* = 6 for further investigation. Figure 3 shows the confusion matrices obtained by all classifiers at the class level. The confusion matrices and other metrics such as recall, precision and *F*-scores at the order and family levels are given in the Supplementary File. At the class, order and family levels, the yeast taxa were handled well by all the classifiers although their sizes were different. For CNN and DBN, the number of wrongly classified sequences at the class and order levels were insignificant. At the family level, the most sequences classified wrongly were classified as *Saccharomycetaceae* (81 by CNN and 50 by DBN). *Saccharomycetaceae* had also the most sequences classified wrongly as other families (21 by CNN and 32 by DBN). This is reasonable as the sequences of *Saccharomycetaceae* were widely distributed as seen in Fig. 1F. For RDP, the most sequences wrongly classified were classified as *Naemateliaceae* at the family level, as *Taphrinales* at the order level, and as *Taphrinomycetes* at the class level. These taxa had the smallest number of sequences in the training dataset. For BLAST, the most sequences wrongly classified were classified as unidentified. This is because these sequences had a similarity score to its best match lower than the optimal threshold predicted for the associated group. Supplementary Fig. 4 shows that at the family and higher taxonomic levels, the recall, precision and *F*-scores obtained by all classifiers were about the same for large groups containing more than ten sequences. However, for small groups containing less than ten sequences, BLAST performed better than the machine learning based approaches. A similar result was also observed for the mold dataset (see Supplementary Fig. 8).

**Figure 4:**
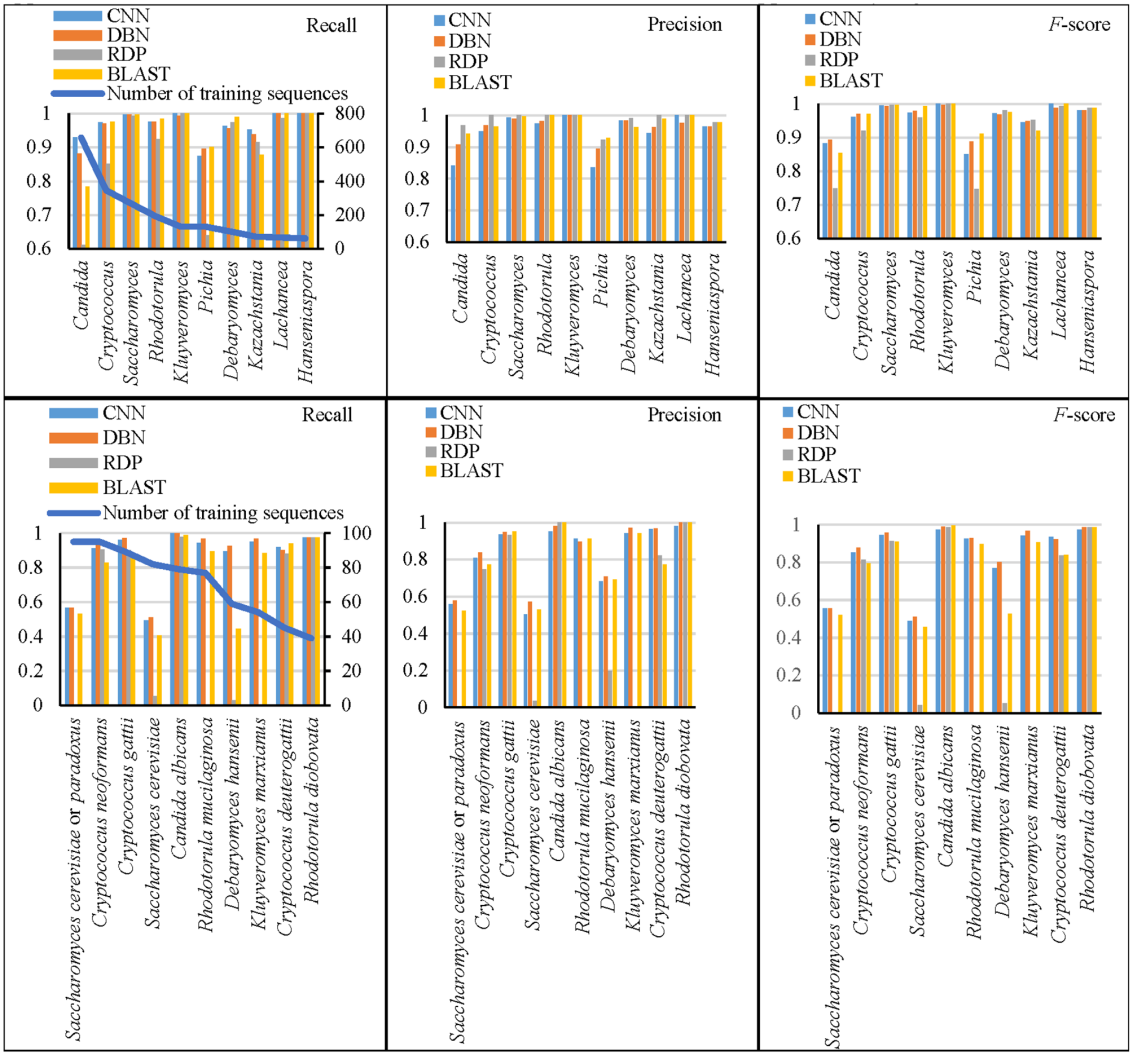
The average recall, precision, and F-scores obtained by different classifiers of the ten species and genera that have the largest number of sequences in the training datasets (ranging from 62 to 657 at the genus level, and ranging from 39 to 95 at the species level) when *k* = 6.

At the genus and species levels where the distribution of the sequence was more equal, the recall, precision, and *F*-scores obtained by all classifiers were about the same. To see which genera and species were not handled well by the classifiers, the average recall, precision, and *F*-scores together with the number of the sequences of the ten largest genera and species were studied (see Fig. 4). At the genus level, *Candida* and *Pichia* had the lowest recall, precision, and *F*-score of less than 90% by all the classifiers. It is because the anamorphs of some *Pichia* species are classified as *Candida* species, which were indistinguishable by ITS. In addition, the species in *Pichia* were reported to have extremely large variations in length^35^ while the genus *Candida* has been suggested for reclassification in^8^. At the species level, only two species *Saccharomyces cerevisiae* or *paradoxus* and *Saccharomyces cerevisiae* were not properly handled by CNN and DBN with a recall, precision, and *F*-score value of ~ 50%. Note that the name *Saccharomyces cerevisiae* or *paradoxus* refers to strains that could not be identified up to species level accurately as *S. cerevisiae* or *S. paradoxus* at the time, and therefore, were named as *Saccharomyces cerevisiae* or *paradoxus*. These species belong to the *Saccharomyces *sensu stricto complex in which the different species can mate and generate viable hybrids, and were known to have extensive differences in genomic and phenotypic variation^36,37^. For BLAST, next to the two *Saccharomyces* species, *Debaryomyces hansenii* was also poorly detected with a low *F*-score of 52%. For RDP, next to the three species mentioned above, *Rhodotorula mucilaginosa*, and *Kluyveromyces marxianus* were handled with an *F*-score of 0. For the mold dataset, the two genera *Chaetomium* and *Acremonium* and the four species *Fusarium oxysporum, Chaetomium globosum*, *Colletotrichum gloeosporioides*, and *Penicillium chrysogenum* were not detected well by all the classifiers (see Supplementary Section 2). It should be noted that these four species are known as species complexes, therefore, the discrepancies in classification are surely a result of ITS being insufficient for species delineation within these complexes. In addition, there could be issues with reference sequence identification, especially for strains that were not ex-types or were identified based solely on morphological characters.

#### Classification probability

To study the minimum probability needed for CNN and DBN to classify fungi, we took the probability scores produced by CNN and DBN with *k* = 6 on the first test dataset for the evaluation. For each interval $[i/100,\;(i + 1)/100]$ with $95 \le i \le 100$, we calculated the number of true and false predictions having a classification probability falling within this interval (Supplementary Fig. 5). The error rate of each interval was calculated as the fraction of false predictions in all predictions for the interval. At the genus and higher taxonomic levels, the number of false predictions were insignificant, compared with the number of true predictions. At the species level, there were several false predictions having a high probability of 0.99 or more. When the maximum error rate was set to 0.1, the minimum classification probability for fungal classification at the species, genus, family, order, class, and phylum levels for CNN and DBN were 0.99.

#### Relation between probability and BLAST similarity scores

To see if classification probabilities were sufficient to assign a sequence to a correct taxonomic group, the relation between classification probabilities and BLAST similarity scores was investigated. The optimal thresholds predicted to classify the current training dataset at the species, genus, family, order, and class levels were 99.3%, 92.2%, 83.2%, 59.4%, and 59.4%, respectively. Supplementary Fig. 6 shows the classification probability and BLAST scores of this dataset obtained by the CNN and DBN classifiers at all taxonomic levels. Interestingly, all classification probability score curves drop after a value of 99%. The percentage of the predictions with a probability score greater or equal than 99% obtained by CNN (DBN) at the species, genus, family, order, and class levels were 64% (46%), 91% (85%), 91% (90%), 92% (97%), and 100% (98%) respectively. Among the predictions by CNN (DBN) having a classification probability of more than 0.99, the percentages of the predictions having a BLAST similarity score greater or equal than the predicted threshold at the species, genus, family, order, and class levels were 92% (95%), 90% (90%), 95% (96%), 100% (100%), and 100% (100%) respectively. The percentages of the predictions by the CNN (DBN) classifier having a BLAST similarity score 10% lower than the predicted threshold at the species, genus, family, order, class, and phylum levels were 0.4% (0%), 0.8% (3.7%), 0% (0.57%), 0% (0%), and 0% (0%) for CNN (DBN) respectively. These results indicate that the CNN and DBN classifiers agreed with the BLAST classification in most of the cases (90–100%). However, at the species and genus levels, even with a high classification probability score of more than 99%, they might still assign the sequences to a taxonomic group with a low BLAST similarity score due to the lack of reference sequences in the training dataset.

### Classifying the “Top 50 Most Wanted Fungi”

The datasets used for the evaluation in the previous section were well curated, in which for each sequence of the test dataset there was always a sequence of the same or closely related taxonomic group in the training dataset. Therefore, the classification results were highly accurate by the machine learning classifiers. We investigated if these classifiers were capable of revealing unidentified sequences from the environmental samples that do not have many reference sequences for training. To this end, we reclassified 2024 most frequently sampled environmental ITS sequences of 1,493 undefined lineages of the “Top 50 Most Wanted Fungi”^31,38^ which were compared with the mold dataset^11^, using the yeast dataset. We hoped to identify a number of yeast sequences from the “Top 50 Most Wanted Fungi”.

**Figure 5:**
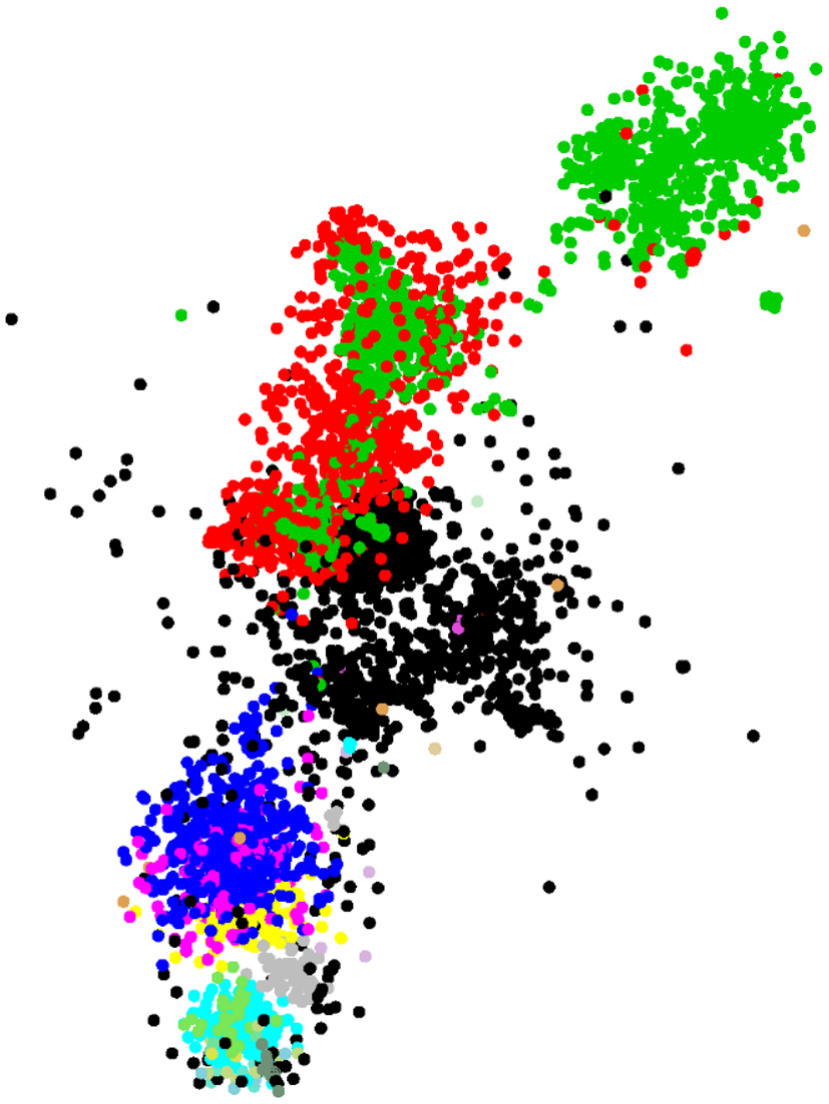
The distribution of the sequences of the yeast dataset together with 2,024 sequences (in black) of the “Top 50 Most Wanted Fungi”. The 730 sequences in red are the sequences of the *Candida* genus. The remaining sequences of the largest order *Saccharomycetales* (1,697) were in green, followed by *Tremellales* (559) in blue, *Sporidiobolales* (305) in cyan, *Trichosporonales* (159) in pink, *Filobasidiales* (122) in yellow, etc.

Figure 5 shows the distribution of the “Top 50 Most Wanted Fungi” sequences (in black) with the yeast sequences. It is interesting to see that the most wanted fungi form a new group lying in between two orders *Saccharomycetales* and *Tremellales*. To avoid the problem of over classifications, at the genus and higher taxonomic levels, the *Candida* sequences were removed from the training dataset as they were distributed widely. In addition, the classified sequences were compared with the barcode sequences of the predicted taxon name using BLAST. Only sequences with a BLAST coverage of at least 300 bp (the minimum length of ITS sequences^11^) and a similarity score higher than or equal to the optimal threshold predicted for this dataset which was 99.4% for species, 93.2% for genus, 84.9% for family, 83.2% for order, and 60.1% for class classification, were assigned to the final group of the classification. For CNN and DBN, *k* was set to 6. We used a probability score of 0.9 for CNN and DBN and the default confidence of 0.8 for RDP to classify the sequences.

Table 3 shows the numbers of sequences of the “Top 50 Most Wanted Fungi” assigned/classified by the BLAST, RDP, CNN, and DBN classifiers at different taxonomic levels. The numbers of sequences assigned and not assigned by both BLAST and RDP, BLAST and CNN, BLAST and DBN with the same name and different names are also given. The CNN classifier classified most of the sequences to the largest group which was *Saccharomyces cerevisiae* or *paradoxus* (35%) at the species, *Pichia* (72%) at the genus, *Saccharomycetaceae* (90%) at the family, *Saccharomycetales* (92%) at the order, and *Saccharomycetes* (72%) at the class level. These groups were distributed widely as seen in “Evaluation of the barcode datasets”. The DBN classifier classified most of the sequences to a highly divergent group, which was *Pichia fermentans* (16%) at the species, *Pichia* (18%) at the genus, *Dipodascaceae* (35%) at the family, *Saccharomycetales* (65%) at the order, and *Agaricostilbomycetes* (71%) at the class level. In contrast with CNN, at a taxonomic level, RDP classified most of the sequences to the smallest group, which were *Candida carvajalis* (78%), *Martiniozyma* (97%), *Carcinomycetaceae* (52%), *Taphrinales* (98%), and *Taphrinomycetes* (99%).

**Table 3:** Number of sequences of the “Top 50 Most Wanted Fungi” assigned/classified (a/c) by BLAST, RDP, CNN, and DBN, and assigned by both BLAST and RDP, BLAST and CNN, BLAST and DBN with the same and different names.

Level	Species	Genus	Family	Order	Class
BLAST	**1**	**17**	**207**	**295**	**337**
RDP (assigned/classified)	0/93	3/630	9/500	13/1775	8/1806
Same assign. by RDP&BLAST	0	3	6	9	8
Diff. assign. by RDP&BLAST	0	0	3	4	0
RDP/BLAST	0	0	0	0	0
BLAST/RDP	1	14	198	282	329
CNN (assigned/classified)	1/281	4/440	87/1546	153/1775	224/1708
Same assign. by CNN&BLAST	1	4	51	121	187
Diff. assign. by CNN&BLAST	0	0	19	17	16
CNN/BLAST	0	0	17	15	21
BLAST/CNN	0	13	137	157	134
DBN (assigned/classified)	0/230	1/335	10/450	50/815	80/1,377
Same assign. by DBN&BLAST	0	1	6	43	68
Diff. assign. by DBN&BLAST	0	0	4	7	12
DBN/BLAST	0	0	0	0	0
BLAST/DBN	1	17	197	245	257

The highest numbers of sequences assigned by the classifiers at all taxonomic levels are highlighted in bold.

BLAST could assign the most sequences of the “Top 50 Most Wanted Fungi”. The numbers of assigned sequences by the machine learning classifiers were much less than the numbers of classified sequences, indicating an obvious problem of over classifications. None of the assigned sequences belonged to the most classified group at all taxonomic levels by RDP, and at species and genus levels by CNN and DBN. The CNN classifier had the highest numbers of assigned sequences, compared with DBN and RDP, which were 100% at the species level, 24% at the genus, 42% at the family, 52% at the order, and 66% at the class level of the sequences assigned by BLAST. At the species level, only BLAST and CNN were able to assign one sequence of GenBank id JX974759 to the species *Rhodotorula sphaerocarpa* with 100% BLAST identity and a coverage of 332 bp to the best match KY104896. At family and higher taxonomic levels, there were 17, 15 and 21 sequences assigned by CNN but not by BLAST. The sequences that were assigned by BLAST but not by CNN, were either belonging to a group that had a small number (less than ten) of reference sequences, or they were classified by CNN with a smaller probability score less than 90%, or they were classified by CNN with a high BLAST identity but a low BLAST coverage (< 200 bp). BLAST and CNN together could assign 1, 17, 224 (11%), 310 (15%), and 353 (17%) sequences of the “Top 50 Most Wanted Fungi” to the species, genus, family, order, and class level, respectively. The assignment of the sequences can be found in the supplementary file namely top50classified.xlsx. In conclusion, to classify a novel dataset that were unrepresented in the training dataset, BLAST classification was the best method, followed by the CNN, DBN, and RDP classifiers.

## Conclusion

This study compared different classification approaches namely CNN, DBN, RDP and BLAST for fungal classification, using two recently released barcode datasets^8,11^. The deep learning neural networks CNN and DBN have been applied for fungal classification for the first time. The result showed that a *k*-mer size of 6 was optimal in terms of accuracy and efficiency for CNN and DBN. The CNN and DBN classifiers worked extremely well on the datasets that had many of the labels present in the training datasets. The CNN classifier outperformed the BLAST, DBN and RDP classifiers at all taxonomic levels in terms of accuracy. Specifically, at the family and higher taxonomic levels, it achieved an extremely high accuracy ranging from 90 to 99%. The DBN classifier did not classify the sequences accurately at the species level. However, at the higher taxonomic levels, it was comparable to the CNN classifier. As for efficiency, it took the machine learning approaches up to two seconds while it was about one minute for BLAST to classify a test dataset on the same computer.

Our study revealed that the fungal genera such as *Candida*, *Pichia*, *Chaetomium*, and *Acremonium* and species such as *Saccharomyces cerevisiae* or *paradoxus*, *Saccharomyces cerevisiae*, *Fusarium oxysporum, Chaetomium globosum*, *Colletotrichum gloeosporioides*, and *Penicillium chrysogenum* were not handled well by all classifiers, suggesting that they are in need for reclassification; or there could be issues with reference sequence identification, especially for strains that were not ex-types or were identified based solely on morphological characters; or ITS is not the best biomarker for their identification. These fungal species are known as species complexes, and therefore, ITS is likely insufficient for species delineating. To improve the prediction at the species level, it might be better to omit species complexes or closely-related species where ITS is insufficient for delineation, as seen in^8,11^.

Although the CNN and DBN classifiers agreed with the BLAST classification in most of the cases, they might still assign the sequences to a wrong taxonomic group even with a high classification probability score due to the lack of reference sequences. The novel dataset “Top 50 Most Wanted Fungi” forming a distinct group lying in between the two orders *Saccharomycetales* and *Tremellales* that was not present at all in the training dataset, highlighted this problem of over classification. After filtering out the over classified sequences, CNN could assign the most sequences, compared with DBN and RDP. BLAST and CNN together could assign 1, 17, 224, 310, and 353 sequences of the “Top 50 Most Wanted Fungi” to the species, genus, family, order, and class level respectively.

The result of the current study will enable us to speed up the taxonomic assignments for the fungal barcode sequences generated at our institute as ~ 70% of them still need to be validated for public release. In addition, it will help to quickly provide a taxonomic profile for metagenomics samples. It also highlights the necessity for publicly-available, authenticated reference sequences, which means supporting ongoing biodiversity sampling efforts. Without reference sequences the best classifier will always fall short.

## Methods

### Predicting optimal thresholds to separate sequences

The optimal thresholds for sequence classification at different taxonomic were estimated using a training dataset. The sequences of the train dataset were compared with each other using BLAST^5^. For each of the obtained local alignments of two sequences, a BLAST score was the percentage of identical matches *s* if the alignment length *l* was greater than 300 bp (the minimum length of ITS sequences^11^). Otherwise it was recomputed as $s \times l/300$. The similarity score of two sequences was the maximum BLAST score of all the local alignments of the two sequences. The sequences were clustered with different thresholds ranging from 0.5 to 1 with a step of 0.001 using the algorithm finding connected components in a graph as this algorithm was shown as one of the most accurate algorithms for DNA sequence clustering^14,32^. The optimal threshold at a taxonomic level was the one producing the highest accuracy (*F*-measure) for clustering^14,39^ compared with the taxonomic classification at the current level. This function is available at https://github.com/vuthuyduong/fungiclassifiers/models/BLAST/trainBLAST.py.

### The classifiers

#### BLAST classification

The test sequences were aligned with the training sequences using BLAST. At a taxonomic level, if the obtained similarity score of a test sequence to its best match exceeded the optimal threshold of this level, the sequence was assigned to the corresponding taxon name of its best match. The implementation of this function can be found at https://github.com/vuthuyduong/fungiclassifiers/models/BLAST/classifyBLAST.py in which BLAST version 2.6.0 was used.

#### The RDP classifier

The RDP classifier^18^ is based on the naïve Bayesian model using a feature space consisting of all possible 8-mer words. For a word *w* and group *G*, a probability score is computed to decide if a member of *G* contains *w*. The probability that a sequence *s* belongs to *G* is computed based on the probabilities of all the words of *s* belonging to a member of *G*. The sequence *s* is assigned to the group giving the highest probability. The RDP classifier was downloaded from https://github.com/rdpstaff/classifier.

#### The CNN classifier

CNNs^25^ consist of two components: convolutional layers and fully connected layers. The convolutional layers are to extract useful features from the input. Each of them consists of convolutional kernels to filter the input, a pooling layer to reduce the number of parameters of the input tensor, and an activation function to determine if a node in the CNN should be activated or not. The fully connected layers in principle are the same as the multi-layer perceptron (MLP) consisting of hidden layers of nonlinearly-activating nodes which takes the output of the convolution layers as its input for classification. The CNN architecture used in this paper was the same as in^28^ which was shown to produce high accuracy for classifying a dataset of simulated 16S DNA bacterial sequences. In particular, two convolutional layers were used with 5 and 10 kernels of size 5, respectively, followed by a max pooling of size 2 and the Rectified Linear Unit (ReLU) activation function. The fully connected layer contained only one hidden layer of 500 nodes with the softmax activation function. Sequences were represented as input vectors of *k*-mer frequencies^40^ of length 4, 6, and 8 to find best *k*-mer for classification in terms of accuracy and run-time performance. The reason of not using higher values for *k* was to avoid computational expenses. As can be seen in^18,41^, small values of *k* were sufficient for DNA barcode classification.

#### The DBN classifier

DBNs^29,30^ are probabilistic generative models, composed of unsupervised networks like Restricted Boltzmann Machines (RBM)^42^ (Hinton 2002) with gradient descent and backpropagation where each sub-network’s hidden layer serves as the visible input layer for the next. Each RBM learns a representation of the input in a lower dimensional space, and in a backward manner, it is possible to obtain an estimation of the probability distribution of the original input. Again, we used the same architecture of the DBN classifier used in^28^ with two hidden layers of 256 units. We increased the number of iterations for backpropagation from 100 to 500, and reduced the dropout rate from 0.2 to 0.1 to increase the accuracy of the DBN classifier on the DNA fungal barcode datasets. For the input of the DBN classifier, sequences were represented as input vectors of *k*-mer frequencies^40^, as for the CNN classifier. Furthermore, the min–max normalization was applied on the *k*-mer frequency vector to scale down the range of data between 0 and 1 to improve the accuracy of the DBN classifier.

### Implementation and experiments

The training, classifying, and evaluation of the BLAST, RDP, CNN and DBN models were implemented in Python 2.7. We used the Keras library (https://www.keras.io) with tensorflow backend for CNN and the source code available at https://github.com/albertbup/deep-belief-network for DBN as used in^28^. The source code of the experiments and datasets are available at https://github.com/vuthuyduong/fungiclassifiers. The benchmark experiments were performed in a high-performance computing (HPC) cluster (64 bit Intel(R)-Xeon(R) Gold 6148 CPU and 16 GB RAM).

## Supplementary information

Supplementary Information 1.

Supplementary Information 2.

## Data Availability

The source code and data are available at https://github.com/vuthuyduong/fungiclassifiers.
