# Supplementary material for: Convolutional neural networks improve fungal classification
Source: Sci Rep. 2020 Jul 28;10:12628. doi: 10.1038/s41598-020-69245-y (PMC7387343; doi:10.1038/s41598-020-69245-y)
Supplement: Supplementary file 1 — Supplementary Information 1. [file 41598_2020_69245_MOESM1_ESM.docx]

Convolutional neural networks improve fungal classification

Duong Vu^1,*^

^1^Westerdijk Fungal Biodiversity Institute, Uppsalalaan 8, 3584CT Utrecht, The Netherlands

# Evaluation on the yeast dataset

This section provides supporting results for the performance of CNN, DBN, RDP, and BLAST on the yeast dataset.


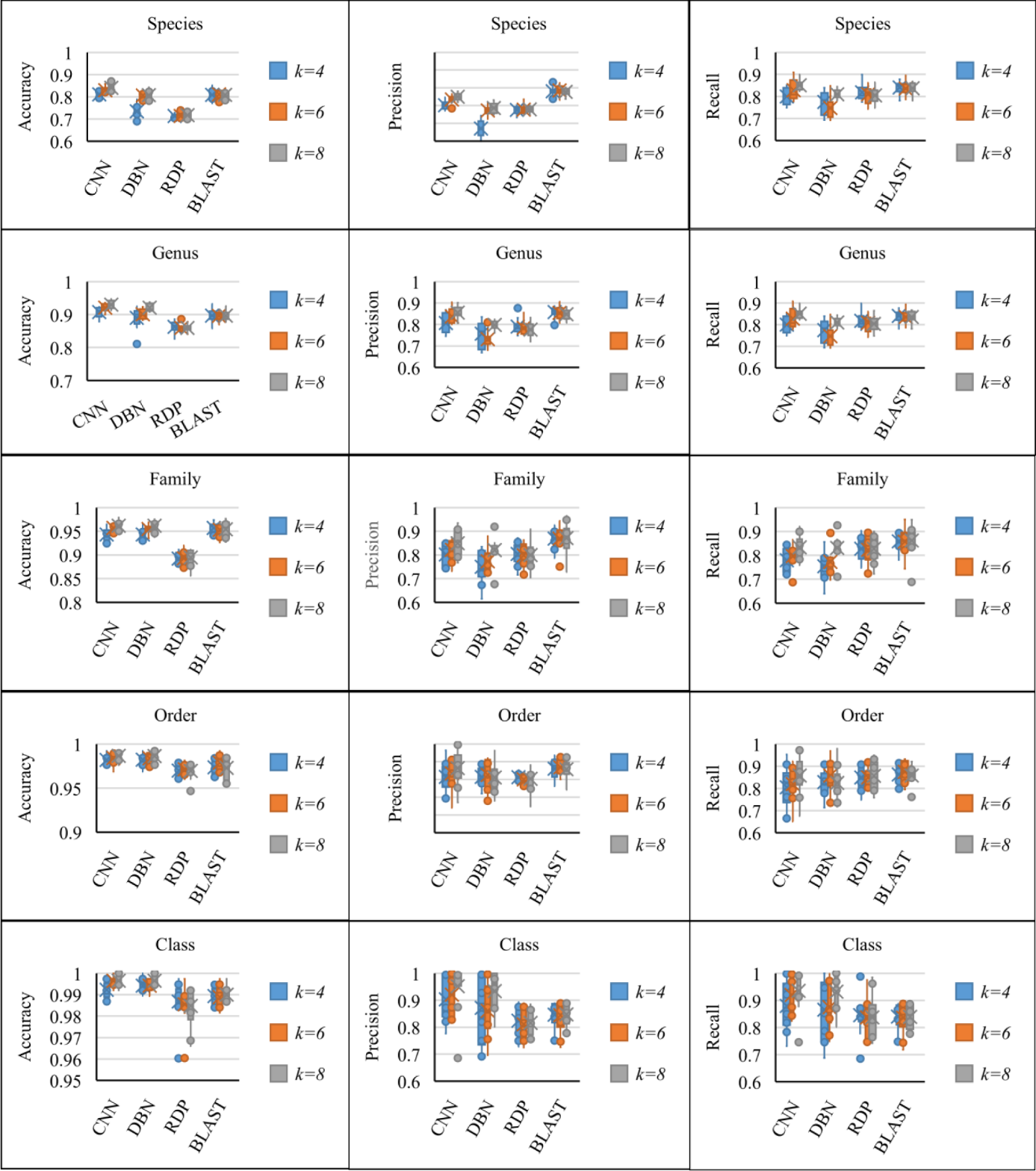
Supplementary Figure 1. The accuracy, precision and recall scores of the yeast test datasets obtained by CNN, DBN, RDP, BLAST at all taxonomic levels with *k*=4, 6, 8.


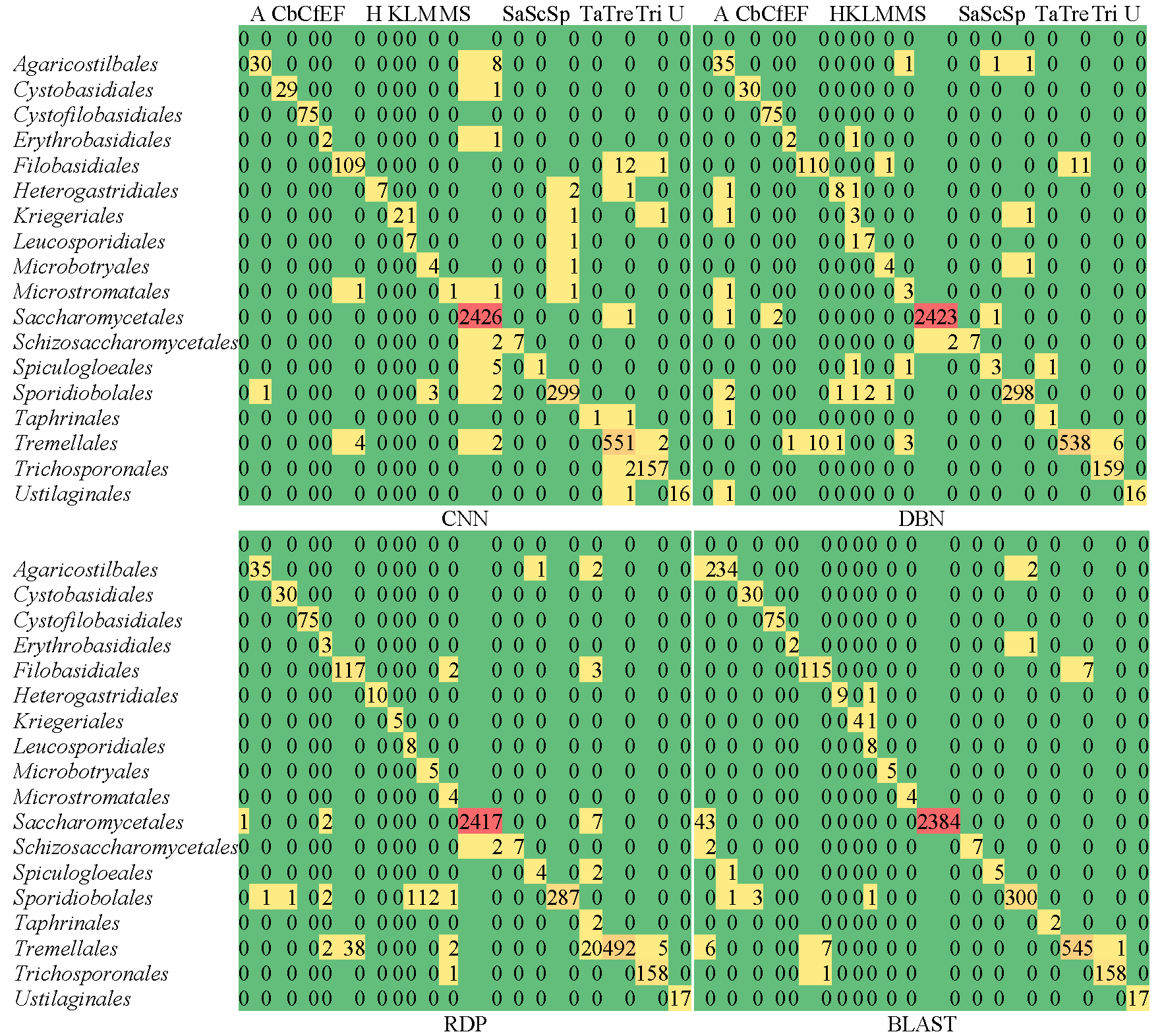
Supplementary Figure 2. The confusion matrices obtained by all classifiers at the order level on the yeast dataset with *k*=6.


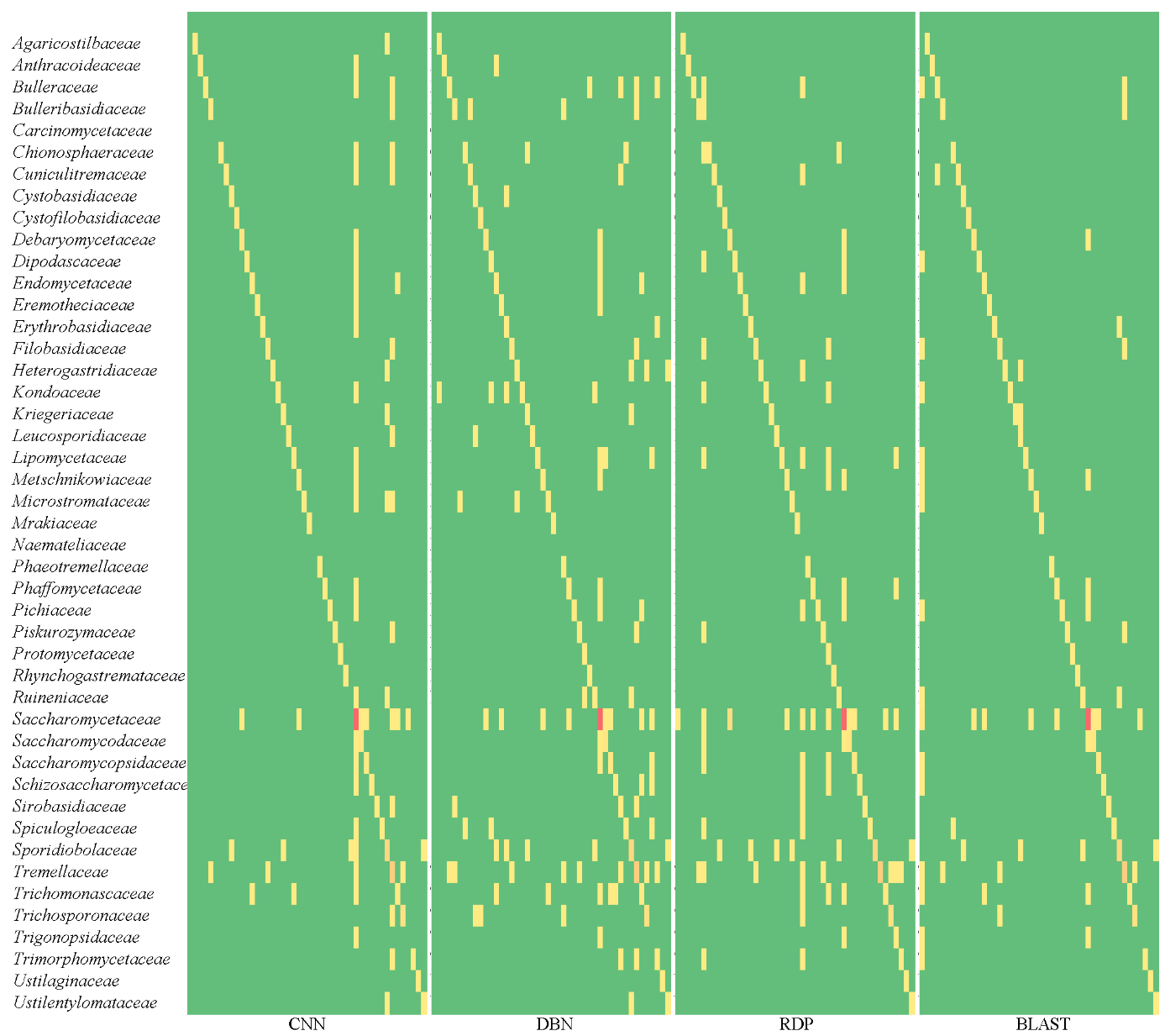
Supplementary Figure 3. The confusion matrices obtained by all classifiers at the family level on the yeast dataset with *k*=6.


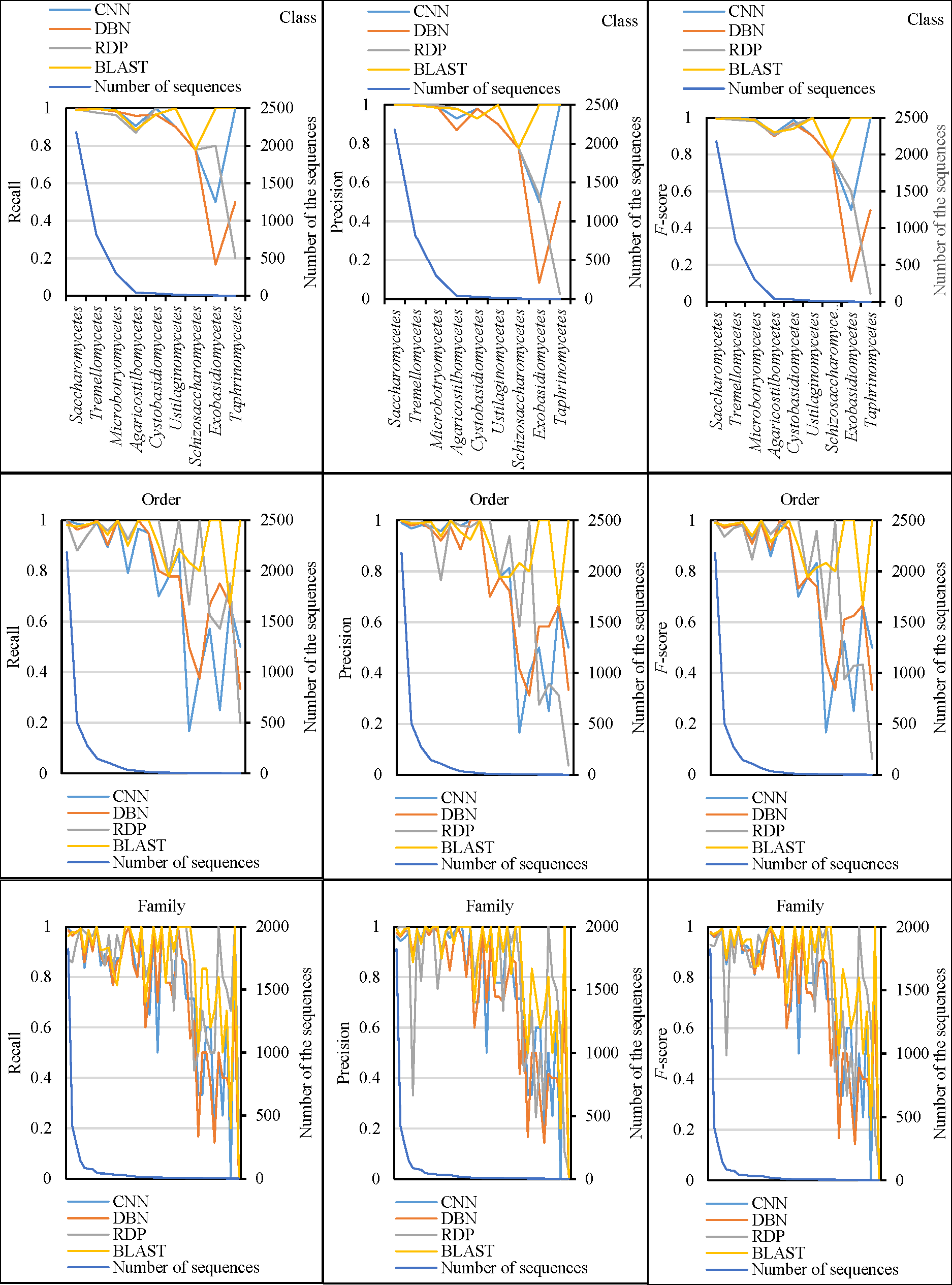


Supplementary Figure 4. The recall, precision, and *F-*scores together with the number of the sequences (on the secondary axis) of each taxon name obtained by all classifiers at the family, order and class levels on the yeast dataset with *k*=6.


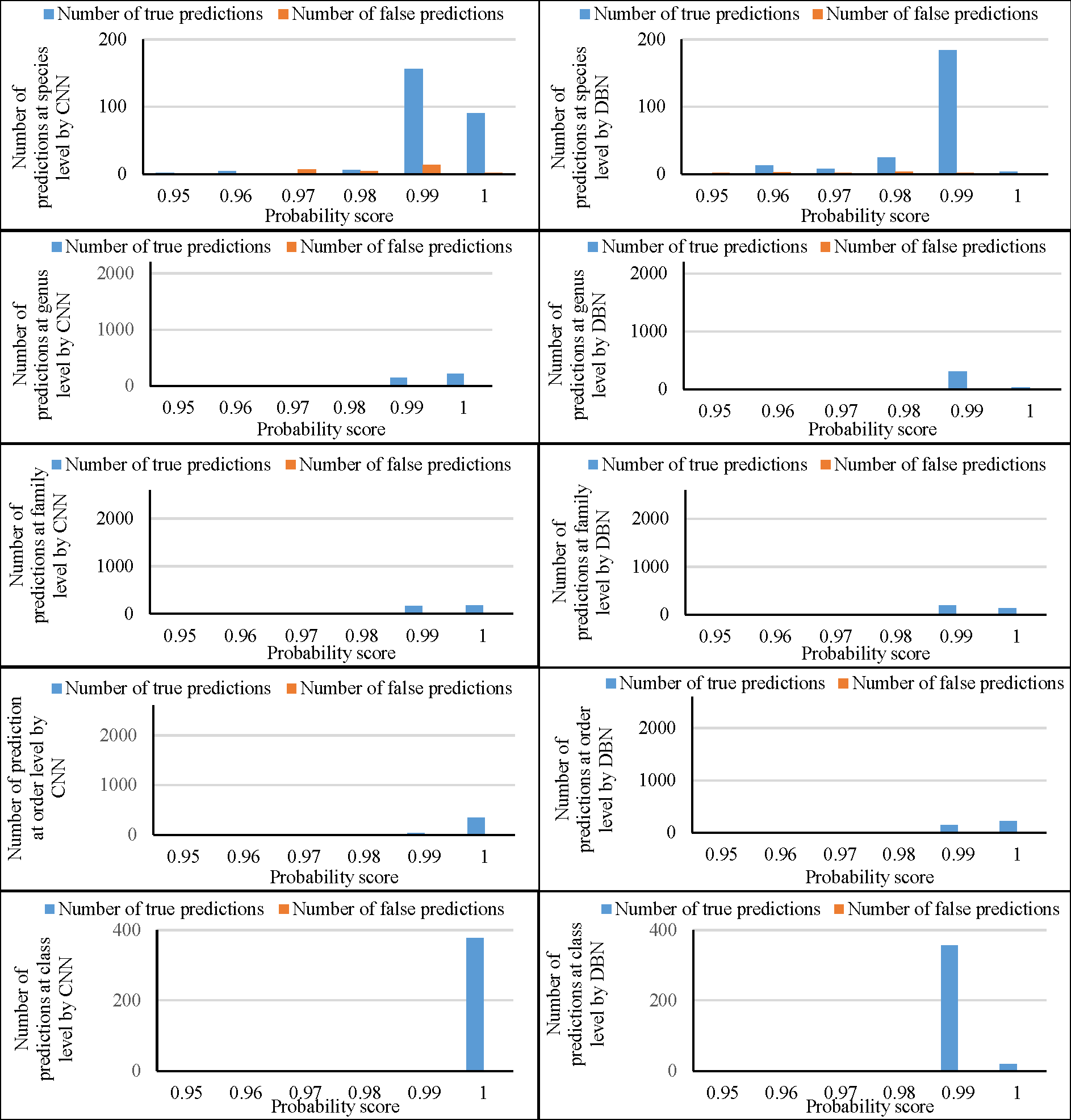


Supplementary Figure 5. The numbers of true and false predictions of the intervals $\left[ i/100,(i+1)/100 \right]$ with $95\leq$*i*$\leq100$, obtained by the CNN and DBN classifiers.


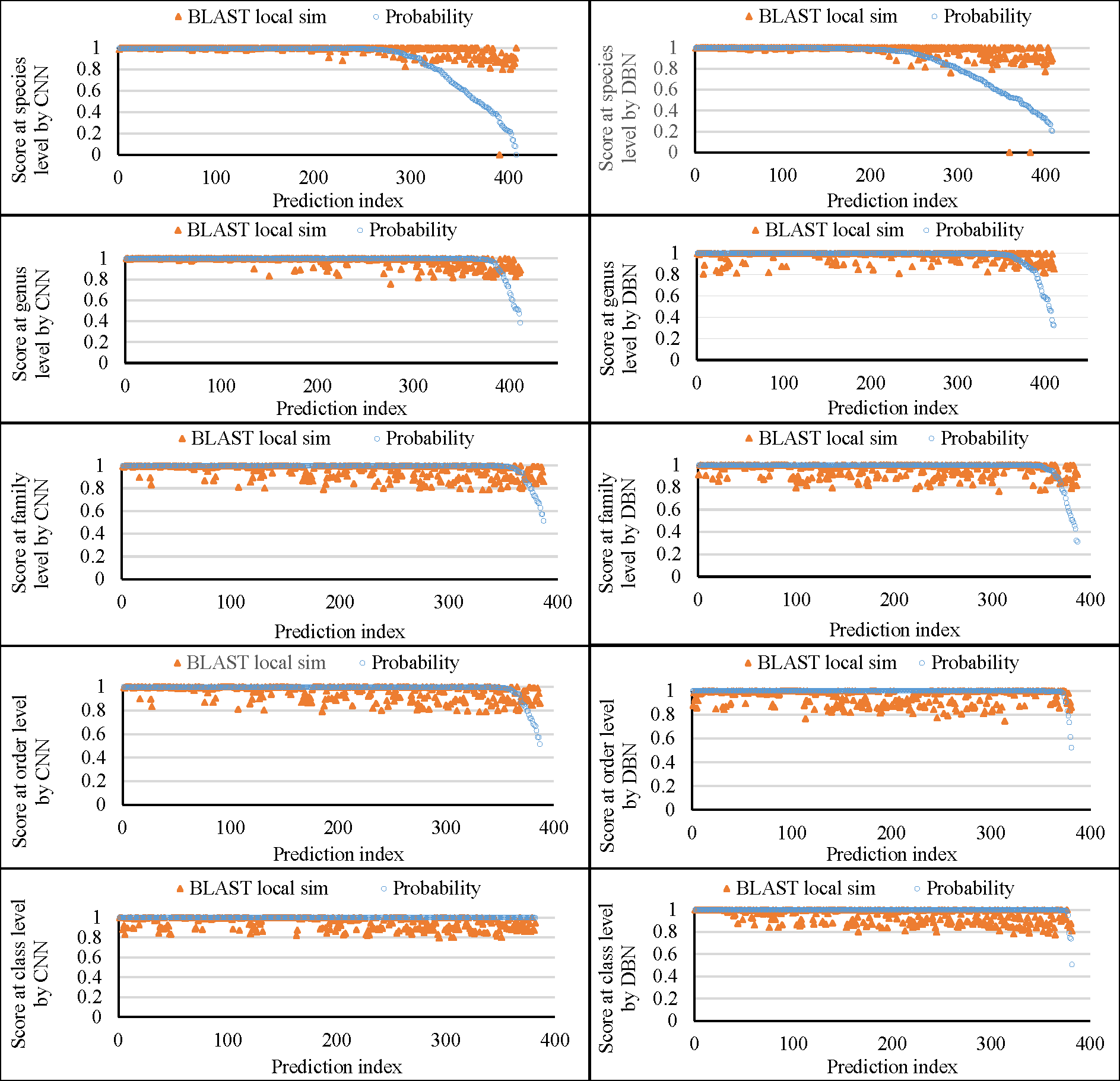
Supplementary Figure 6. The classification probability and BLAST similarity scores of the yeast dataset produced by the CNN and DBN classifiers.

# Evaluation on the mold dataset

In this section, we applied CNN, DBN, RDP and BLAST classification on the released filamentous fungal (mold) dataset representing 40 % cultured molds consisting 11,715 ITS barcode sequences of 6,042 species, 1,619 genera, 346 families, 114 orders, and 36 class [1] (<https://www.ncbi.nlm.nih.gov/bioproject/PRJNA422523>). Like the yeast dataset, the mold sequences were not distributed equally either, in which 17 %, 25 %, 22 %, 24 %, and 67 % of the sequences at the class, order, family, genus and species level were singleton (see Supplementary Fig. 7A). The first five largest groups at the class, order, family and genus levels consisted of 91 %, 52 %, 30 %, and 18 % of the sequences of dataset, respectively. The median similarity scores of sequences within a group at each level were also varied, specifically at higher taxonomic levels, as seen in Supplementary Fig. 7B. Supplementary Fig. 7C shows the best thresholds predicted to separate the sequences that were 0.998 with *F*-measure of 75 % at the species level, 0.968 with *F-*measure of 60 % at the genus level, 0.965 with *F*-measure of 57 % at the family level, 0.952 with *F*-measure of 52 %, and 0.946 with *F*-measures of 49 %. The optimal threshold at the species level was high because it was known in [1] that ~17 % mold species could not be separated by ITS. At higher taxonomic levels, the best *F*-measures obtained were low, as the sequences were not equally distributed as seen in Supplementary Fig.7D.


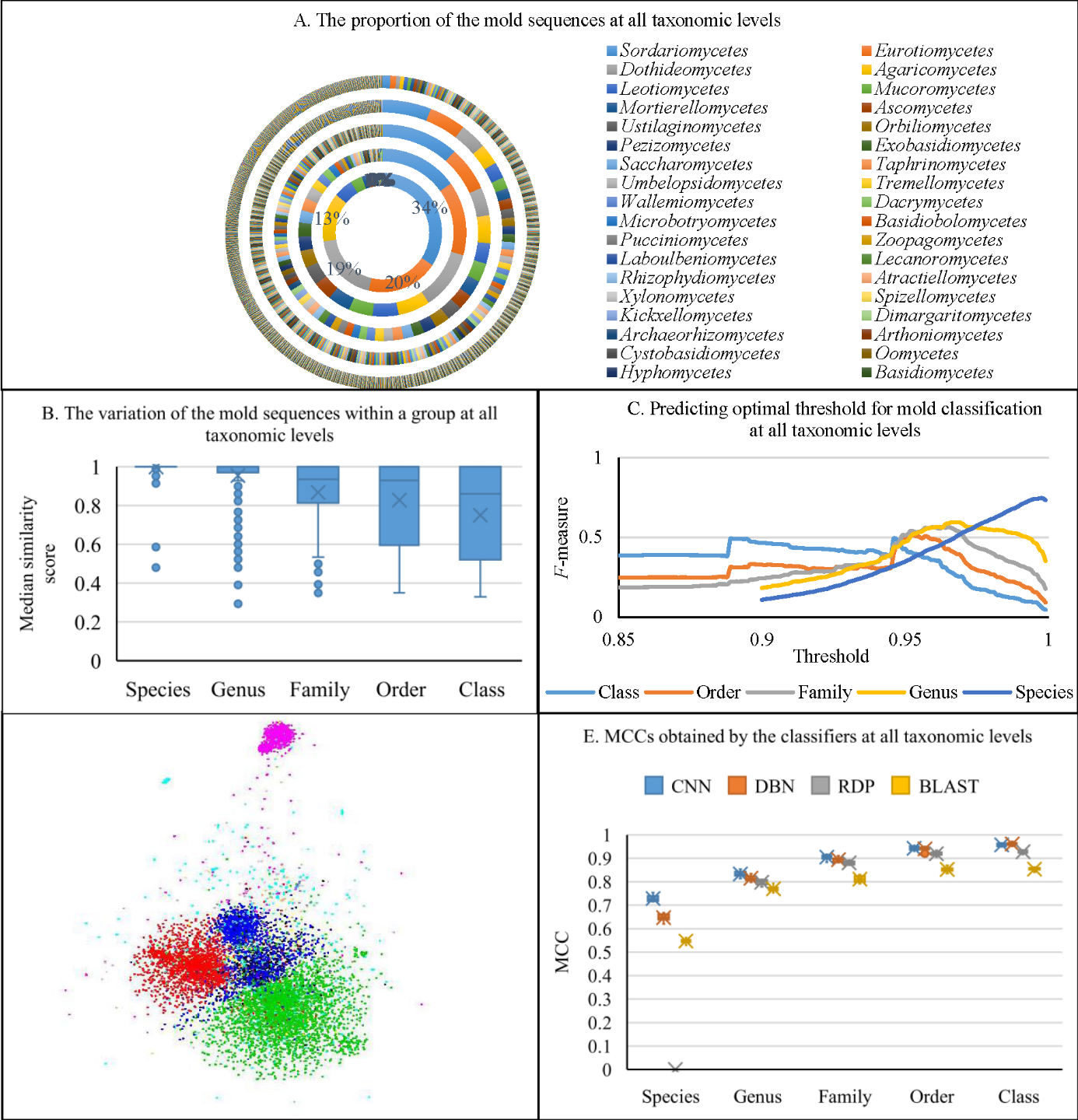
Supplementary Figure 7. A: The proportion of mold sequences at all taxonomic levels. The smallest ring represents the class level, followed by the order, family, genus and species levels. B: The variation of the median similarity scores of the mold groups at all taxonomic levels. C: Predicting optimal thresholds for the mold dataset using a series of thresholds (between 0.85 and 1, with a step of 0.001) at all taxonomic levels. D: The distribution of the mold dataset in which sequences of the same class have the same color. The four groups in green, red, blue and pink represent ITS sequences of *Sordariomycetes* (3,899), *Eurotiomycetes* (2,221), *Dothideomycetes* (2,150), and *Agaricomycetes* (1,511), respectively. The numbers in brackets are the numbers of the sequences in the current group. The coordinators of the sequences were generated using fMLC [2]. The sequences were visualized using the rgl package in R (https://r-forge.r-project.org/projects/rgl/). E: The MCCs of the mold test datasets obtained by all the classifiers at different taxonomic levels.

To evaluate the qualities of the classifiers, the dataset was also split into two datasets, the training and test datasets, in a tenfold cross-validation procedure, like for the yeast dataset. On average, there were 10536 (90 %) sequences of 5,643 (93 %) species, 1,551 (96 %) genera, 338 (98 %) families, 111 (97 %) orders, and 35 (97 %) classes for training and 1171 sequences for testing of which 394 (3.4 %), 68 (0.58 %), 8 (0.07 %), 3, and 1 sequences in the test dataset had no labels in the train dataset at the species, genus, family, order, and class level respectively. For CNN and DBN, *k* was set to 6. The RDP classifier did not work at the species level due to memory constraints. Supplementary Fig. 7E shows the MCCs obtained by different classifiers on the mold dataset at all taxonomic levels. At the species level, the average MCCs obtained by CNN, DBN, RDP and BLAST were 0.73, 0.65, 0 and 0.54 respectively. At the genus level, they were 0.83, 0.81, 0.8 and 0.77. At the family level, they were 0.91, 0.89, 0.88 and 0.81. At the order level, they were 0.94, 0.94, 0.92 and 0.85. At the class level, they were 0.96, 0.96, 0.93 and 0.85. Again, the CNN classifier outperformed the other classifiers. The BLAST classification produced the lowest MCCs on this dataset, because the high optimal thresholds predicted excluded all the sequences lying in the border of the groups.

To see how all the taxa were handled by the classifiers, the recall, precision and *F*-scores of each group at different taxonomic levels obtained by all classifiers were studied (see Supplementary Fig. 8 and Fig. 9). Like the yeast dataset, at the family and higher taxonomic levels, the CNN and DBN also produced the same *F*-score as BLAST for classes containing more than ten sequences. For small classes containing less than ten sequences, BLAST worked better. At the genus and species levels where the distributions of the sequences were more equal, the recall, precision, and *F*-scores obtained by all classifiers were about the same. Supplementary Fig. 8 shows the recall, precision and *F*-score of the first ten largest genera and species obtained by all the classifiers. It can be seen that the genera *Chaetomium* and *Acremonium* had an *F*-score less than 90 % by all the classifiers. The sequences within these genera were highly divergent. The median and minimum similarity scores of *Chaetomium* were 94.1% and 84.4 %. For *Acremonium*, they were 72.6 % and 51.7 %, respectively. The four species *Fusarium oxysporum, Chaetomium globosum*, *Colletotrichum gloeosporioides*, and *Penicillium chrysogenum* had an *F*-score of less than 80% by all the classifiers. It is because they had a low precision score as the species *Fusarium oxysporum* and *Chaetomium globosum* had a number of varieties in the training dataset while *Colletotrichum gloeosporioides* and *Penicillium chrysogenum* were known to share ITS sequences with their closely related species [3, 4].


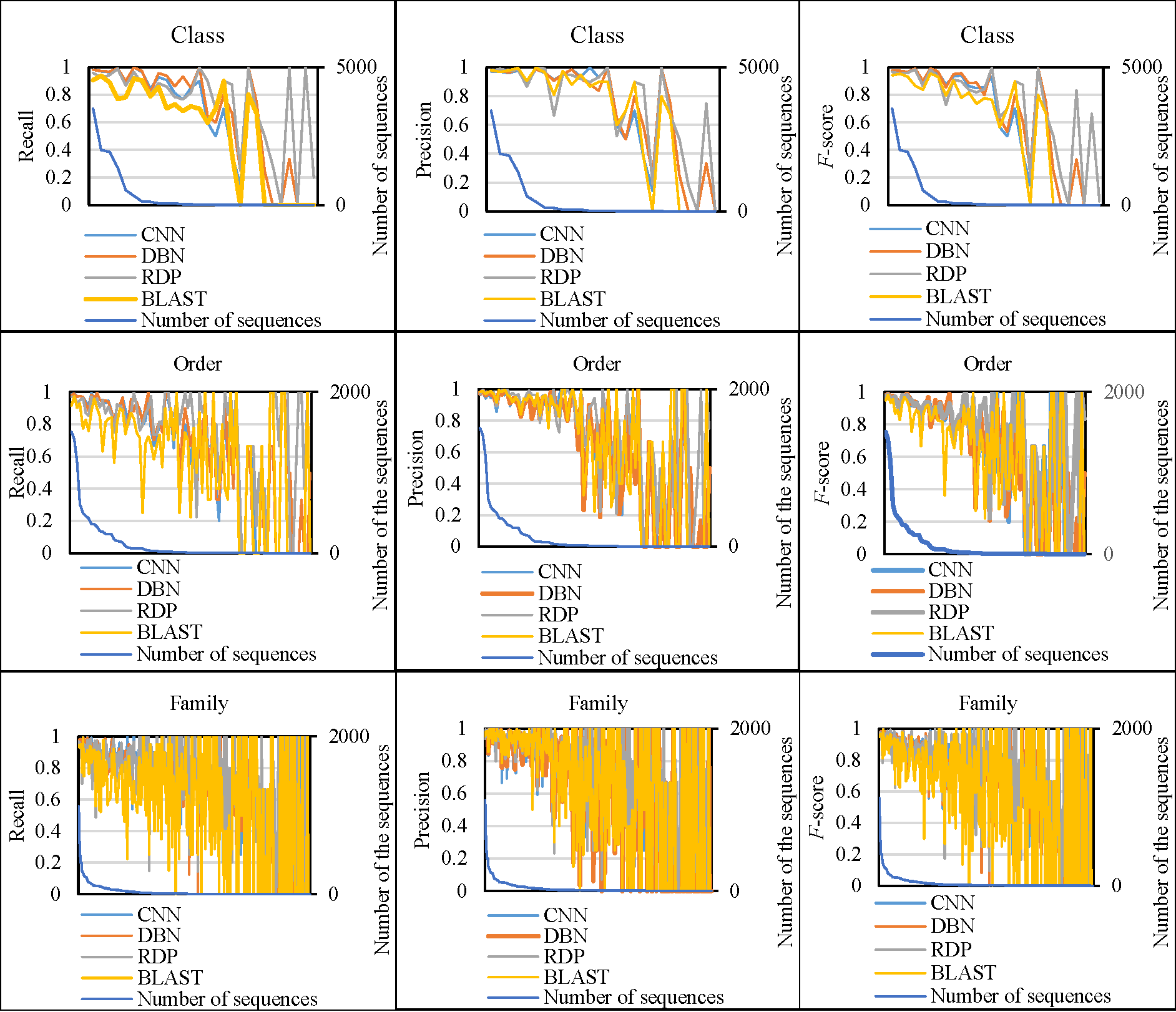


Supplementary Figure 8. The recall, precision, and *F-*scores together with the number of the sequences (on the secondary axis) of each taxon name obtained by all classifiers at the family, order and class levels on the mould dataset with *k*=6.
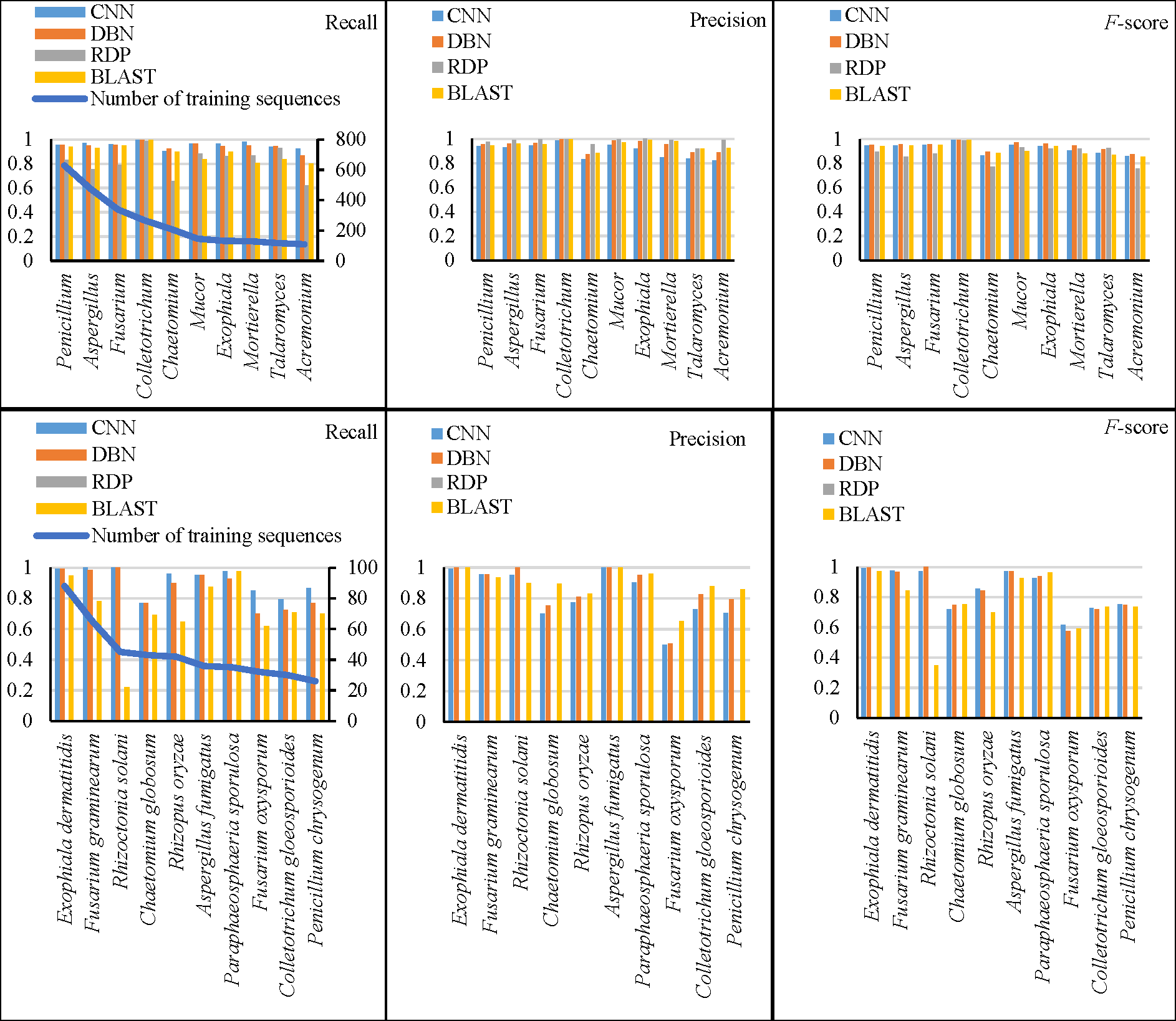
Supplementary Figure 9. The average recall, precision, and *F*-scores obtained by different classifiers of the ten largest mold genera and species (with the number of training sequences ranging from 109 to 629 at the genus level, and from 26 to 88 at the species level) when *k*=6.

References

1. Vu, D., Groenewald, M., de Vries, M., *et al.* (2019). Large-scale analysis of filamentous fungal DNA barcodes reveals thresholds for species and higher taxon delimitation. *Studies in Mycology* **92**, 135-154.
2. Vu, D., Georgievska, S., Szöke, S., *et al.* (2018). fMLC: Fast Multi-Level Clustering and Visualization of Large Molecular Datasets. *Bioinformatics* **34**, 1577–1579.
3. Houbraken, J., Frisvad, J.C., Seifert, K.A., *et al.* (2012). New penicillin-producing Penicillium species and an overview of section Chrysogena. *Persoonia* **29**, 78–100.
4. Weir, B.S., Johnston, P.R., Damm, U. (2012). The Colletotrichum gloeosporioides species complex*. Studies in Mycology* **73**, 115–180.
